# Pelvic Fractures—An Underestimated Problem? Incidence and Mortality Risk after Pelvic Fracture in Austria, 2010–2018

**DOI:** 10.3390/jcm11102834

**Published:** 2022-05-17

**Authors:** Martina Behanova, Judith Haschka, Berthold Reichardt, Hans-Peter Dimai, Heinrich Resch, Jochen Zwerina, Roland Kocijan

**Affiliations:** 1 Ludwig Boltzmann Institute of Osteology at Hanusch Hospital of OEGK and AUVA, Trauma Centre Meidling, 1st Medical Department Hanusch Hospital, 1140 Vienna, Austria; judith.haschka@osteologie.lbg.ac.at (J.H.); jochen.zwerina@osteologie.lbg.ac.at (J.Z.); roland.kocijan@osteologie.lbg.ac.at (R.K.); 2 Austrian Social Health Insurance Fund, Österreichische Gesundheitskasse, 7000 Eisenstadt, Austria; berthold.reichardt@oegk.at; 3 Division of Endocrinology and Diabetology, Department of Medicine, Medical University of Graz, 8036 Graz, Austria; hans.dimai@medunigraz.at; 4 Metabolic Bone Diseases Unit, School of Medicine, Sigmund Freud University Vienna, 1020 Vienna, Austria; heinrich.resch@bhs.at; 5 2nd Department of Internal Medicine, St. Vincent Hospital Vienna, 1090 Vienna, Austria

**Keywords:** pelvic fracture, epidemiology, osteoporosis, mortality, Austria

## Abstract

(1) Background: Pelvic fractures (PFs) are related to osteoporosis, and represent a serious individual and socioeconomic burden. (2) Methods: We examined age- and sex-standardised incidence rates (SIRs) of PF, along with rates of all-cause overall and one-year mortality among patients with PF. We compared the mortality rates between PF patients and a matched fracture-free cohort. Patients ≥50 years old in Austria hospitalised with PF in 2010–2018, along with their dates of death, were recorded. (3) Results: We identified 54,975 patients with PF, of whom 70.9% were women. Between 2010 and 2018 the SIR of PF increased in men by 10.0%—from 125.3 (95% Confidence Interval 118.9–132.0) to 137.8 (95% CI 131.8–144.0) per 100,000—and in women by 2.7%—from 218.7 (95% CI 212.0–225.6) to 224.7 (95% CI 218.3–231.3) per 100,000. The one-year post-PF mortality rate was higher in men than in women (13.0% and 11.1%, respectively; *p* < 0.001). Pelvic fracture patients aged ≥65 had an elevated mortality risk (Hazard Ratio 1.75, 95% CI 1.71–1.79, *p* < 0.001) compared to controls. (4) Conclusions: There is a clear increase in the incidence of PF in the elderly population, with a greater increase in men over time. Pelvic fracture itself contributes to increased mortality in individuals aged 65 and above.

## 1. Introduction

Pelvic fractures (PFs) are underestimated osteoporotic fractures [1], leading to serious individual and socioeconomic burdens [2,3]. In comparison to hip fractures, which have been thoroughly investigated, PFs have not been as rigorously studied. There are not many nationwide register-based studies that report the incidence and trends of pelvic fracture (PF) in the general population [4,5,6,7,8,9]. These studies show increasing rates of PF in the elderly population, and suggest that fragility fractures of the pelvis will cause challenges for the health and social care systems in the future. As with hip fractures, pelvic fractures are associated with high hospitalisation and mortality rates [7,10], similarly to hip fractures. It has been shown that female patients aged 75 and above are associated with the highest odds of discharge to an inpatient facility [9]. Male patients above 65 years old had excess mortality in the first four months after PF compared to women [11]. The risk of sustaining a subsequent fracture is also high. Patients aged 54–70 years who sustained PFs were almost five times more likely to sustain a subsequent PF [12]. The overall mortality of pelvic ring fractures decreased over the 22-year study period in Germany from 9.3% to 3.8%, although with higher male than female mortality [13]. The odds of complications and mortality after PF in patients aged above 80 years were higher compared to 65–79-year-olds in the US [14].

While absolute numbers of hip fractures have increased due to the aging of the population, age-standardised incidence rates are levelling off or even declining in numerous countries. This has also been observed in Austria, where the age-standardised incidence of hip fractures has been declining in the Austrian population aged ≥50 years [15,16]. In contrast, age-standardised rates of PF have increased in recent decades; moreover, the observed numbers of PF are even higher than predicted. In Finland, the projected case load of first osteoporotic PF was 1400 per 100,000 by the year 2010 in the population aged ≥60 [5], but a recent study reported 1508 cases per 100,000 [8].

Austria has a social health insurance system that covers 99.9% of all people in the country [17]. It stands among countries with the lowest self-reported unmet medical needs in the European Union (EU). Although expenditure growth is similar to the EU average, Austria continues to spend substantially more on inpatient care than most countries, and has high numbers of physicians and hospital beds [18]. With regard to fracture rates, Austria ranks among countries with the highest incidence rates worldwide for hip fractures [15,16], proximal humerus fractures [19], and distal forearm fractures [20].

The epidemiological situation with respect to pelvic fractures in Austria has not yet been thoroughly analysed. The main objectives of this nationwide study were to calculate age- and sex-specific incidence rates of PF between 2010 and 2018, to determine the rates of all-cause overall and one-year mortality among patients with PF, to compare mortality between PF patients and a matched fracture-free cohort in Austria, and to assess trends in these indicators.

## 2. Materials and Methods

### 2.1. Austrian Social Health Insurance Fund

Prior to 2020, each of Austria’s nine federal states (*Bundesländer*) had its own health insurance fund, through which the majority of private sector employees were insured. The regional health insurance fund at which the workplace was located was responsible for the health insurance. In the case of retired citizens, the affiliation was based on their place of residence [17]. Public sector employees had the Austrian Civil Service Insurance. Mining and railway sector employees had their own health insurance. There were two forms of social security for the self-employed, for farmers and freelancers. In January 2020, nine of these health insurance funds were merged to form the Austrian Health Insurance Fund. The public and railway sector were merged and the two Social Securities for the Self-employed representing three health insurance providers.

### 2.2. Study Design and Study Population

This study was a nationwide retrospective register-based observational study that included all patients in Austria aged ≥50 from nine former health insurance providers, representing 76% of the insured population in Austria. The cutoff point of 50 years was based on the fact that the incidence of low-traumatic fractures significantly increases from the age of 50 years [21]. The lifetime risk for a 50-year-old woman to suffer an osteoporotic fracture is 50%. Thus, international guidelines recommend an assessment of fracture risk in patients above 50 years who have sustained a previous fracture or who have risk factors for osteoporotic fractures [22]. Furthermore, fracture liaison services (FLSs)—systematic approaches to prevent further fracture risk after a fracture—are typically applied to patients aged >50 years [23,24].

**Figure 1:**
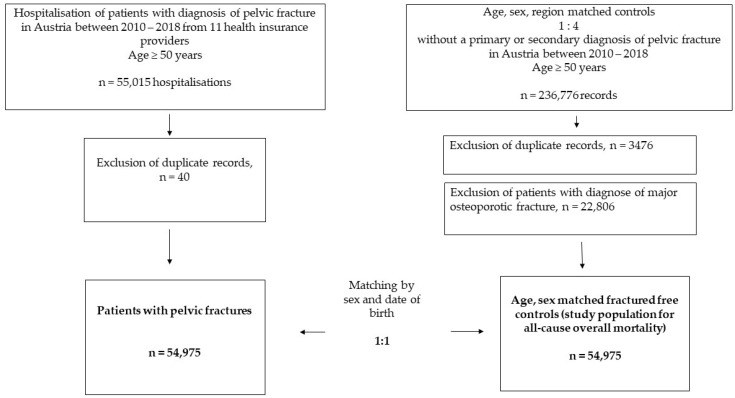
Flowchart of the study population.

Patients’ pseudonymised individual-level data were obtained from social insurance authorities and the Federal Ministry of Labour, Social Affairs, Health, and Consumer Protection in Austria. Patients’ data were drawn from the entire 50-year-old population of Austria insured by one of the nine health insurance providers, which was 2,213,973 in 2010 and 2,674,610 in 2018. Data on age and sex distributions for the Austrian population in 2010–2018 for the calculation of standardised incidence rates were obtained from the *Statistik Austria* [25]. See Figure 1 for a flowchart of the study design and patient selection.

### 2.3. Cohort of Patients with Pelvic Fracture

We identified patients aged 50 years or older with inpatient hospitalisation for a main or side diagnosis of a PF between 1 January 2010 and 31 December 2018. A PF event was defined by the following ICD-10 codes: S32.1 (fracture of os sacrum), S32.2 (fracture of os coccyges), S32.3 (fracture of os ilium), S32.4 (fracture of acetabulum), S32.5 (fracture of os pubis), S32.6 (fracture of ischium), S32.81 (multiple fractures of the pelvis with disruption of the pelvic ring), and S32.89 (fracture of other parts of the pelvis). In line with the latest evidence, we deliberately did not distinguish between high and low trauma in this population at high risk of fractures. Recently published international statements recommend that physicians stop classifying fractures as high- or low-trauma fractures, because high-traumatic fractures are also often associated with low BMD and osteoporosis [26]. Thus, high-traumatic fractures are also associated with low BMD and, even more importantly, with subsequent fracture risk [12]. Consequently, all fractures, regardless of type of trauma, are potentially osteoporotic, and should be evaluated in patients aged > 50 years [26].

### 2.4. Controls without Major Osteoporotic Fractures

Controls were obtained from the same data source of the insured population as the PF cohort, and they could be also patients without inpatient stay. Controls were individually matched by date of birth and sex to PF cases, and had to be alive on the index date of their respective PF case. The matching ratio was 1:1. Controls were free of major osteoporotic fractures defined by the following ICD-10 codes: S72 (fracture of hip), S22 (fracture of spine), S42 (fracture of humerus), S52 (fracture of forearm), and S32 (fracture of pelvis).

### 2.5. Incidence Rates of Pelvic Fractures

Our primary outcome measure was incidence of PF, defined as the number of patients who had a diagnosis of PF during the investigation’s timeframe (2010–2018). A new incident PF diagnosis was defined as hospital discharge where the PF was recorded for the first time in that period, as either the main diagnosis or a side diagnosis. Individual patients were counted only once if the patient had sustained more than one PF within a reported year.

### 2.6. Mortality

Our secondary outcomes were one-year all-cause mortality, overall all-cause mortality, and mortality risk between PF and controls.

The index date in the PF cohort was defined as the earliest PF event between 1 January 2010 and 31 December 2018. Time to death was calculated from the index date until the date of death or to the end of follow-up on 8 March 2020.

### 2.7. Recording of Covariates

Data on demographics and vital status were available for PF cases and controls. Comorbidity conditions recognised on hospital discharges were available for PF patients and controls after the index date.

### 2.8. Statistical Analyses

For PF incidence, we calculated annual age- and sex-specific standardised incidence rates (SIRs) and 95% confidence intervals (95% CIs) for 2010–2018. We used direct standardisation by using the mean population of Austria between 2010 and 2018 as the standard population size. We calculated the incidence rate ratio for sexes for each year as the incidence rate of women divided by the incidence rate of men for that particular year.

We calculated one-year mortality and overall all-cause mortality rate. The follow-up period covered the time between the index date and the end-of-follow-up date (8 March 2020 for overall mortality, 365.25 days for one-year mortality) or the patient’s death.

All analyses were stratified by sex and two age groups (50–64 and above 65 years old) because of the significant interaction of the groups (PF vs. control) by sex and by age for one-year as well as overall mortality.

For the one-year mortality analysis we used the Kaplan–Meier estimator to show the probability of surviving up to one year after PF and in disease-free matched controls. All patients were censored after 365.25 days. The all-cause mortality rate was calculated by dividing the number of deceased patients by the total person-years of follow-up.

In addition, we calculated 7- and 30-day all-cause mortality in PF patients, and assessed the proportion of patients who sustained PF at the same time as hip fracture.

To see the effect of PF on mortality, we compared mortality between PF patients and the age- and sex-matched fracture-free cohort. The proportional hazards (PH) assumption for Cox proportional hazard regression was assessed by visual examination of Log (−Log) plots (Supplementary Materials, Figures S1 and S2). The PH assumption was violated only in women aged 65 years and above; therefore, we did not proceed with the Cox regression analysis, but performed logistic regression—crude and adjusted. For the remainder, we proceeded with Cox proportional hazard regression and calculated hazard ratios (HRs) with 95% confidence intervals (CIs)—crude and adjusted.

In the mortality analysis, we adjusted for age at the index date and the weighted Elixhauser index. The list of comorbidities for computation of the Elixhauser index can be found in Supplementary Table S1.

We calculated weighted Elixhauser scores for both PF cases and controls following the method of Quan et al. [27], using the R package “comorbidity” [28]. We stratified the weighted version of the grouped Elixhauser index using the algorithm of Van Walraven et al. [29], into four categories (<0, 0, 1–4, and ≥5). Van Walraven et al. developed a scoring system for the Elixhauser index, using the regression coefficients for each of the 30 comorbidities from a multivariate logistic regression model predicting in-hospital mortality [29]. The rationale behind choosing the Elixhauser comorbidity system was the fact that it slightly outperformed the Charlson system at adjusting for comorbidity, especially in fractured patients [30].

Differences in mean age at PF in the observed period were calculated by ANOVA. Differences in mean EHI index between PF and controls were assessed using the independent-samples Mann–Whitney U test. Differences in the proportion of deceased patients between PF and controls were calculated using Pearson’s chi-squared test.

We summarised the absolute numbers of patients with a diagnosis of PF per fracture location.

### 2.9. Sensitivity Analysis

We performed a sensitivity analysis by excluding all patients with metastatic cancer and solid tumours without metastases and lymphoma to determine whether these conditions might have affected outcomes in mortality.

Statistical analyses were conducted using IBM SPSS Statistics for Windows, version 27 (IBM Corp., Armonk, NY, USA), and R version 3.5.2 [31]. Annual incidence of PF was depicted with MS Excel (Microsoft Office Professional Plus, 2019).

## 3. Results

**Figure 2:**
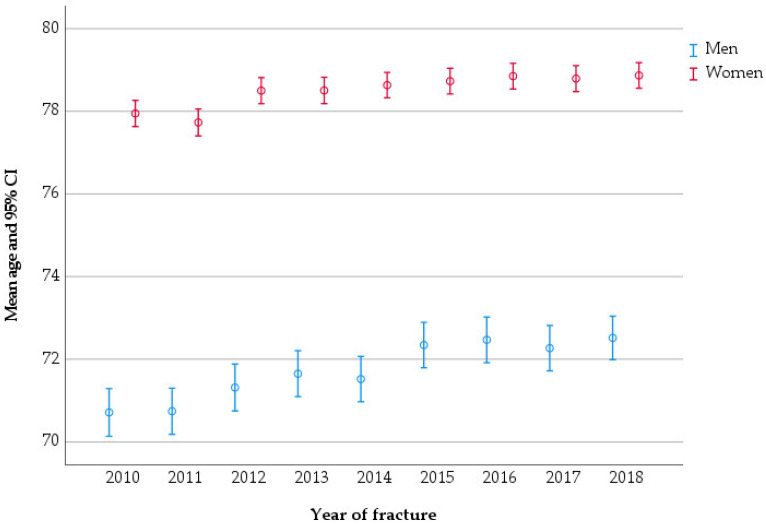
Mean age of patients at pelvic fracture, stratified by the year of fracture.

A total of 54,975 patients aged ≥ 50 years sustained 55,015 pelvic fractures between 2010 and 2018. Of these patients, 16,022 (29.1%) were men, with an average age of 72.0 years (SD 12.0), and 38,953 (70.9%) were women, with an average age of 78.5 years (SD 10.6). The average age of pelvic fracture patients changed over the studied period; in men, it increased from 70.7 in 2010 to 72.5 in 2018 (*p* < 0.001), while in women it increased from 78.0 to 78.9 (*p* < 0.001) (Figure 2).

In men, the median age for sustaining a PF was 73 years (IQR 62–81), whereas in women it was 81 years (IQR 72–86).

There were 54,975 sex- and age-matched fracture-free controls. They had lower mean Elixhauser comorbidity scores (6.5 and 8.2, respectively; *p* < 0.001), and a lower proportion of deceased (27.2% and 38.2%, respectively; *p* < 0.001) (Table 1). There were significant differences in all of the Elixhauser comorbidities between the cohort of PF patients and their controls. PF patients had higher prevalence of almost all comorbidities than controls, apart from metastatic cancer (4.5% and 3.7%, respectively; *p* < 0.001) and solid tumours without metastases (14.8% and 12.8%, respectively; *p* < 0.001) (Supplementary Table S1).

### 3.1. Age-Standardised Incidence Rate

**Figure 3:**
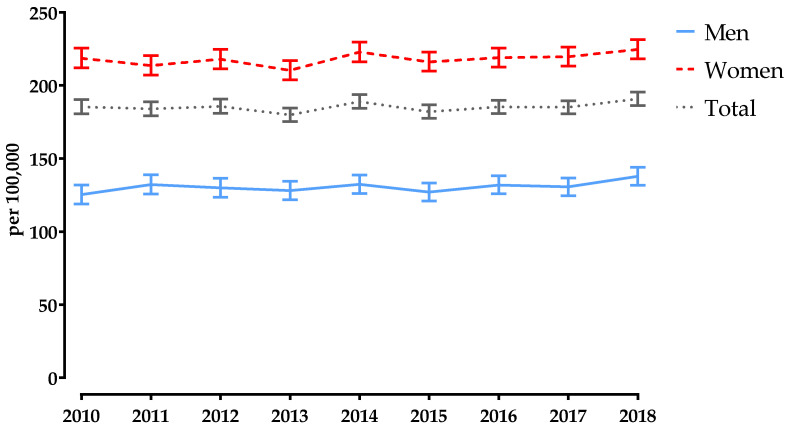
Annual age-standardised incidence rates of pelvic fractures in Austria, stratified by sex, for 2010–2018. Bars represent 95% confidence intervals.

Between 2010 and 2018, the SIR of PF increased in both sexes (Supplementary Table S2). For men, it increased by 10.0%, from 125.3 (95% CI 118–132.0) to 137.8 (95% CI 131.8–144.0) per 100,000, while for women it increased by 2.7%, from 218.7 (95% CI 212.0–225.6) to 224.7 (95% CI 218.3–231.3) per 100,000 (Supplementary Table S2 and Figure 3). Overall, the SIR increased by 2.9%, from 185.4 (95% CI 180.6–190.3) to 190.8 (186.3–195.4) per 100,000 (Supplementary Table S2 and Figure 3). In 2010, the SIR of PF was 1.7 times higher in women than in men, but in 2018 this ratio declined to 1.6 (Supplementary Table S2).

### 3.2. Mortality

There were significant differences in one-year mortality by sex and age group between PF patients and controls. In the age group of 50–64 years old, controls had higher one-year mortality than PF patients (12.4% and 3.4%, respectively), with control women having the highest mortality, at 15.3%. In the age group of 65 years old and above, PF patients had higher one-year mortality than controls (13.4% and 12.0%, respectively), with PF men having the highest mortality, at 16.9% (Table 2).

**Figure 4:**
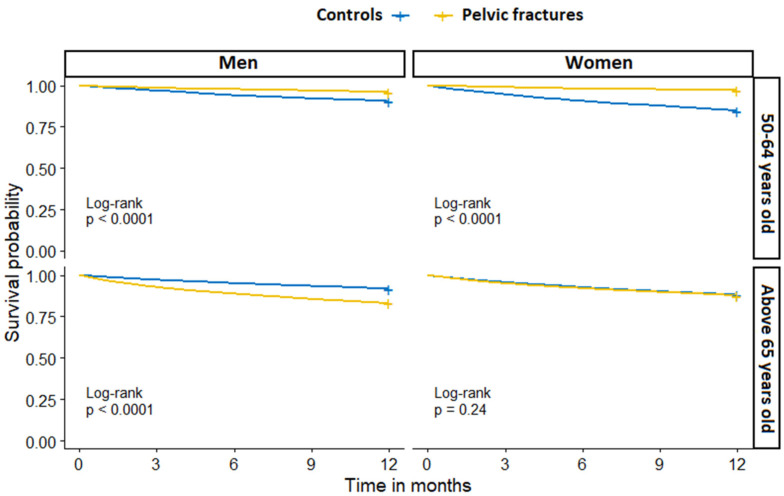
One-year all-cause mortality in patients with pelvic fracture and fracture-free controls, stratified by sex and age group.

The one-year survival probability between PF and controls stratified by sex and age group is presented in Figure 4. 

The overall mortality rate in the age group of 50–64 years old was 20.0 per 1000 person-years (PY) for PF patients, and 69.1 per 1000 PY for controls. The reverse was observed in the age group of 65 years old and above, with a mortality rate of 109.7 per 1000 PY in PF patients, and of 66.0 per 1000 PY in controls (Table 3).

**Figure 5:**
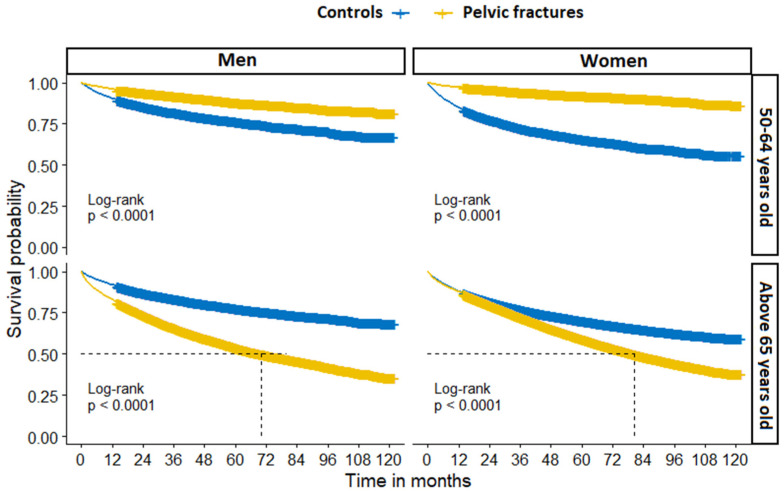
Overall all-cause mortality in patients with pelvic fracture and fracture-free controls, stratified by sex and age group.

There were significant differences in overall survival probability by sex and age group between PF patients and controls (Figure 5).

Crude Cox proportional regression analysis showed a significant association of PF with higher mortality risk, particularly in men, in the age group above 65 years old (HR 2.44, 95% CI 2.33–2.57). After adjusting for age and EHI, the hazard ratio decreased (HR 1.36, 95% CI 1.29–1.43) (Table 3). Similarly, women in this age category with PF had higher odds of dying than fracture-free controls (OR 1.65, 95% CI 1.62–1.69).

Regarding the location of PF, we observed that os sacrum, os coccyges, pubis, ischium, and multiple fractures were more frequent in women than in men, whereas fractures of the os acetabulum and ilium were common in both sexes. In men, the most frequent types of PF were general fracture of the pelvis, fracture of the pubis, and fracture of the acetabulum. In women, the leading subcodes were fracture of the pubis, general fracture of the pelvis, and fracture of the sacrum (Supplementary Table S3).

The results of 7- and 30-day all-cause mortality in PF patients are shown in Supplementary Table S4. The proportion of patients who sustained PF at the same time as hip fracture was 0.6% (n = 352).

### 3.3. Sensitivity Analysis

A sensitivity analysis that excluded all patients (in PF cases and controls, n = 34,935) with metastatic cancer, solid tumours without metastases, or lymphoma did not change the results of one-year nor overall mortality. In the age group of 50–64 years old it was 2.4% in PF men vs. 11.6% in control men, and 1.4% in PF women vs. 15.9% in control women. In the age group of 65 years old and above it was 15.3% in PF men vs. 9.8% in control men, and 11.3% in PF women vs. 12.8% in control women (results not shown). The HR for men in the age category of 50–64 years old was 0.22 (95% CI 0.19–0.25), while for the age group of 65 years old it was 1.68 (95% CI 1.58–1.79). After adjustment for age and EHI, the hazard ratio decreased to 1.49 (95% CI 1.40–1.60) (results not shown).

## 4. Discussion

In this nationwide study, we examined age- and sex-standardised incidence rates of PF in an Austrian population aged ≥ 50 years between 2010 and 2018. Additionally, our study sought to analyse the rates of all-cause overall and one-year mortality among patients with PF, and to compare mortality rates between PF patients and a fracture-free matched control cohort.

We observed that the incidence of PF increased over time in both men and women. Overall, the age-standardised incidence rates increased by 2.9%. The PF rates increased with age, and were most frequently observed around the age of 80 years. PFs were more frequent in women than in men. However, we observed that the women–men ratio in the annual SIR decreased over time, indicating an increasing volume of PFs in men. There are several possible factors that may influence an increase in PF and a decrease or plateau in hip fracture incidence in Austria. We hypothesise that one of those factors could be a change in the body mass index (BMI) of the Austrian population. A study that assessed trends in the prevalence of obesity in the Austrian population in the period between 1983 and 2014 found that the strongest increase in the prevalence of obesity was among women ≥75 years old (13.3% annual change) and in men between 55 and 74 years (10.9%) [32]. Higher BMI has been shown to be protective of bone health, thus mitigating the fracture risk [33]. However, no clear conclusion regarding the relationship between obesity and fracture risk could be drawn, due to the lack of site-specific studies of obesity. We surmise that it is likely that better diagnostics of osteoporosis, heightened awareness, and a better reporting/coding system of PF might play a role.

There are a few studies that have examined the incidence of PF; however, they differ in their sources of data, study designs and reporting, study populations and settings, age of study population, and observational time, thus making it difficult to directly compare their results. Our rates of PF based on in-hospital PF fractures were lower in comparison to a German study that included inpatient and outpatient PFs. They reported an age-standardised incidence rate of 224 per 100,000 in a population above 60 years old [34], with higher rates in women (287 per 100,000). By contrast, Austria’s rates were higher than those reported in Finland (where the rate in the population aged ≥65 was 169 per 100,000) [8] and in the UK (40 per 100,000) [35]. The latter study comprised individuals aged 50 or older, but used only ICD-9 coding for PF, which might partially explain the low rates.

There is evidence that fragility fractures among the elderly population are treated outside trauma centres, making it difficult to ascertain a precise epidemiology of PF. For example, a Dutch study that used data from their National Trauma Registry (2008–2012) reported an incidence of 57.9 per 100,000 in the population aged ≥65 years [36]. In contrast, another study from the Netherlands, which used data from the medical registration (1986–2011), reported an overall age-adjusted incidence rate of 71.4 per 100,000 in a population aged ≥65 years in 2011 [6]. Moreover, a study by Prieto-Alhambra et al. pointed out an underreporting of fractures in medical reports from primary care providers [7]. Nevertheless, differences in incidence rates may be partially attributable to structural differences between healthcare systems, but methodological differences in the studies may also result in discrepancies between findings. However, all studies on PF show increasing trends of PF over time. Differences in age and sex structure over time do not explain the increasing rates of PF in our study. This could be seen from the rates in direct standardisation. Direct standardisation involves applying the rates of disease observed in the study group of people to a ‘standard’ population. More likely, increasing rates of PF are related to increasing life expectancy. Correspondingly, age-related problems such as osteoporotic bone and sarcopenia can result in falls and fragility fractures. The higher observed incidence rates of PF may be also due to advances in diagnostic management of PF by (early) detection of PF. This increasing trend of PF is worrying for several reasons. First, patients with PF often require prolonged frequent care, which increases healthcare costs. A recent study showed that PF patients represent very high utilisation costs after index fractures [3]. Second, the patients’ quality of life after PF is decreased [37]. Post-fracture care after PF in older people requires long periods of immobilisation, along with repeated outpatients’ visits and rehabilitations [2]. Ultimately, longer periods of immobilisation increase the risk of complications, such as cardiopulmonary and thromboembolic events [38], decline in muscle mass and, as a result, a higher risk of fall and subsequent fractures [12].

Evidence shows that one-year mortality after PF ranges from 9.5% to 27% [39,40,41]. With our finding of 11.6%, Austria is positioned on the lower end of the scale. The overall one-year cumulative mortality was 13.9% in Spain [7]. Overall mortality after PF based on the German Pelvic Trauma Registry was 3.8% [13], with male mortality being higher, at 7.6%.

In the present study, we observed sex and age differences in mortality after PF. Men aged ≥65 years had higher one-year post-PF mortality than women. The higher mortality rate in men may be partially explained by their inferior health status as assessed by the Elixhauser index. Furthermore, in the literature there is evidence that the type of treatment (conservative or surgical) might influence the outcome as well. It has been reported that women had a significantly lower mortality rate after conservative treatment compared to men. At the same time, there were no sex differences after surgical treatment [13].

We observed that PF patients had a higher mortality hazard ratio than non-fractured controls. In line with this, a smaller study by Reito et al. found that both men and women with PF had a higher mortality risk than the general population [42]. In our study, it was difficult to determine whether the observed higher mortality was driven by the PF itself or by differences in pre-index comorbidity status. Although we did not use the baseline EHI index, but rather the index from the follow-up period, this may still reflect the additive nature of chronic conditions to mortality after PF.

Unadjusted one-year mortality after hip fracture in women and men in Austria was 18.6% and 24.5%, respectively [43]. In the present study, the one-year post-PF mortality was markedly lower than previously reported post-hip-fracture mortality, but still higher for men than for women. PFs, together with HFs, significantly contribute to premature mortality among the elderly population, regardless of their low- or high–trauma origin. It has been shown that hip fracture is an independent risk factor for long-term mortality in otherwise-healthy elderly individuals, and that increased post-hip-fracture mortality is not purely a result of comorbidities [44].

Although we did not differentiate between high- and low-traumatic fractures in the present study, based on recent reports, in the fractured patients above 50 years old, osteoporotic bone status could be assumed. Evidence shows that PF, independent of trauma, is associated with an increased subsequent fracture risk of the femur and the pelvis [12]. Moreover, PFs are known to be frequently involved in high-traumatic multiple fractures. Associations with fractures of the lumbar or thoracic spine, thorax, and femur have also been reported. In contrast, low-traumatic PFs are accompanied by forearm, humerus, and femur fractures. In patients with low-traumatic fractures of the lumbar and thoracic spine, PFs can often be observed [45].

In contrast to hip fractures, treatment of PFs is usually non-operative. However, the 90-day mortality risk and the readmission rate were reported to be similar for pelvic fractures and surgically treated hip fractures [42].

### Study Strengths and Limitations

Our study has important strengths. We present for the first time data on the incidence and mortality risk of PF in Austria, representing the Central–Eastern European region. Data were available for nine years, giving long-term information on this underreported osteoporotic fracture type. By using unique pseudonyms for patients, we were able to identify hospital-based readmissions and discharges of the same diagnosis, so as to exclude them and to calculate the first PFs in the observed period. Calculation of the readmission rate due to PF was not the aim of this study. On the other hand, the limitation of this study is that only patients treated in hospitals were included. As shown in a recent work by Muschitz et al., the estimated volume of ambulatory-treated PFs in Austria is nearly twice that of hospital-based treated cases [21]. We were limited by our data to clearly answering the question of whether post-pelvic-fracture mortality is attributable to pre-existing comorbidities or to the fracture itself. We lacked causes of death in our dataset, and were only able to calculate all-cause mortality. Another limitation resulting from the register-based study design is that we did not have information on the PF-free intervals before the index dates. By counting multiple PFs per patient in different years, bias may have occurred. However, the proportion of these patients amounts to less than 0.1% of the total fractures (concerning 8 men and 32 women in the observed period), and would not overestimate or affect the results of the incidence rates of PFs.

Next, data on lifestyle factors, socioeconomic situations, history of falls, frailty, and detailed clinical aspects of the fractures (e.g., X-ray confirmation, bone mineral density measures) were not available. Furthermore, we lacked data on the individual patients’ treatment regimes, be they conservative or surgical. Associated injuries were also not present in our dataset.

The studied patients were hospitalised due to different diseases, and were followed up for other hospital-based diagnoses, suggesting more severe conditions compared to an outpatient population. Additionally, 39.2% of controls did not have any record of hospital-based diagnoses during the investigated period. We acknowledge that the confounding factors regarding comorbidities of patients were not thoroughly explored in our study, and this is one of its main limitations.

## 5. Conclusions

Due to rising life expectancy and, thus, comorbidities, the incidence of PF is increasing. This has been found not only for the Austrian population—representing the Central–Eastern European population—but also in other countries. Considering the increased morbidity, mortality, hospitalisation rate, and resultant high socioeconomic costs after PF, clinicians should be aware of PF.

## Figures and Tables

**Table 1:** Characteristics of patients with pelvic fracture and fracture-free controls.

		Pelvic Fracture			Controls		*p*-Value ^a^
	All n = 54,975	Men, n (%) 16,022 (29.1)	Women, n (%) 38,953 (70.9)	All n = 54,975	Men, n (%) 16,022 (29.1)	Women, n (%) 38,953 (70.9)	
Age, mean (SD)	76.6 (11.4)	72.0 (12.0)	78.5 (10.6)	76.6 (11.4)	72.0 (12.0)	78.5 (10.6)	1
Age group, n (%)							1
50–64	9552 (17.4%)	4873 (30.4)	4679 (12.0)	9552 (17.4%)	4873 (30.4)	4679 (12.0)	
65–79	19,549 (35.6)	6279 (39.2)	13,270 (34.1)	19,549 (35.6)	6279 (39.2)	13,270 (34.1)	
≥80	25,874 (47.1)	4870 (30.4)	21,004 (53.9)	25,874 (47.1)	4870 (30.4)	21,004 (53.9)	
ECI score, mean (SD)	8.2 (9.5)	8.8 (10.0)	8.0 (9.2)	6.5 (8.4)	7.1 (8.9)	6.3 (8.2)	<0.001
ECI, n (%)							
<0	2978 (5.4)	609 (3.8)	2369 (6.1)	1682 (3.1)	315 (2.0)	1367 (3.5)	
0	15,908 (28.9)	5107 (31.9)	10,801 (27.7)	12,090 (22.0)	2992 (18.7)	9098 (23.4)	
1–4	5433 (9.9)	1428 (8.9)	4005 (10.3)	3874 (7.0)	1063 (6.6)	2811 (7.2)	
≥5	30,620 (55.7)	8866 (55.3)	21,754 (55.8)	15,781 (28.7)	4052 (25.3)	11,729 (30.1)	
Not known/missing	36 (0.1)	12 (0.1)	24 (0.1)	21,548 (39.2)	7600 (47.4)	13,948 (35.8)	
Deceased, n (%)	21,018 (38.2)	5947 (37.1)	15,071 (38.7)	14,973 (27.2)	3548 (22.1)	11,425 (29.3)	<0.001
Zero follow-up time, n (%)	964 (0.9)	427 (44.3)	537 (55.7)	NA	NA	NA	
Follow-up time in months ^b^, median (Interquartile range IQR)	43.9 (22.7–73.4)	43.2 (21.5–74.7)	44.2 (23.2–73.1)	44.3 (22.9–72.6)	47.4 (25.1–77.0)	42.9 (22.0–71.0)	0.64

^a^*p*-Values for differences between all pelvic fracture cases and controls. ECI: Elixhauser comorbidity index, NA: Not applicable. ^b^ In patients with follow-up time ≥ 1 day.

**Table 2:** One-year mortality after pelvic fracture and in matched fracture-free controls, stratified by age group and sex; n = number of deaths.

	Pelvic Fractures			Controls		
Age Group	All	Men	Women	All	Men	Women
50–64	3.4% (n = 324)	4.0% (n = 194)	2.8% (n = 130)	12.4% (n = 1181)	9.6% (n = 467)	15.3% (n = 714)
65+	13.4% (n = 5951)	16.9% (n = 1826)	12.2% (n = 4125)	11.0% (n = 4996)	8.1% (n = 899)	12.0% (n = 4097)
Total	11.6 (n = 6275)	13.0% (n = 2020)	11.1% (n = 4255)	11.2 (n = 6177)	8.5% (n = 1366)	12.4% (n = 4811)

**Table 3:** Mortality rates (per 1000 person-years) and hazard ratios (HRs) for the association between pelvic fracture and all-cause mortality in a cohort of pelvic-fractured patients and age- and sex-matched fracture-free controls, stratified by age group and sex.

			Overall Mortality					
Age Group	Sex		Deaths	Follow-Up	Mortality Rate per 1000 PY	Model 1	Model 2	Model 3
50–64	Men	Controls	1136	21,095.9	53.8	Reference	Reference	Reference
		PF	587	24,554.9	23.9	0.46 (0.42–0.51) ***	0.47 (0.42–0.51) ***	0.32 (0.28–0.35) ***
	Women	Controls	1527	17,443.7	87.5	Reference	Reference	Reference
		PF	393	24,535.8	16.02	0.20 (0.18–0.23) ***	0.20 (0.18–0.23) ***	0.19 (0.17–0.21) ***
	Total	Controls	2663	38,539.7	69.1	Reference	Reference	Reference
		PF	980	49,090.7	20.0	0.31 (0.29–0.33) ***	0.31 (0.29–0.34) ***	0.25 (0.23–0.27) ***
65+	Men	Controls	2412	48,513.7	49.7	Reference	Reference	Reference
		PF	4933	39,508.0	124.9	2.44 (2.33–2.57) ***	2.66 (2.53–2.79) ***	1.36 (1.29–1.43) ***
	Women	Controls	9898	137,874.1	71.8	Reference	Reference	Reference
		PF	14,141	134,288.6	105.3	1.78 ^1^ (1.72–1.83) ***	1.79 ^1^ (1.74–1.85) ***	1.15 ^1^ (1.10–1.19) ***
	Total	Controls	12,310	186,387.8	66.0	Reference	Reference	Reference
		PF	19,074	173,796.6	109.7	1.65 (1.62–1.69) ***	1.75 (1.71–1.79) ***	1.15 (1.12–1.17) ***

^1^ Results of logistic regression analysis; the numbers represent odds ratios. *** *p* < 0.001. Model 1: crude, Model 2: adjusted for age at index date, Model 3: adjusted for weighted EHI (continuous variable).

## Data Availability

The data presented in this study are available upon request from the corresponding author.

## Supplementary Materials

The following supporting information can be downloaded at: https://www.mdpi.com/article/10.3390/jcm11102834/s1, Supplementary Table S1: Elixhauser comorbidities in a cohort of patients with pelvic fractures and their age- and sex-matched fracture-free controls; Supplementary Table S2: Age- and sex-specific crude (CIRs) and standardised incidence rates (SIRs) of pelvic fracture per 100,000 in Austria between 2010 and 2018; Supplementary Table S3: Absolute number of patients with a diagnosis of pelvic fracture, per fracture location; Supplementary Table S4: 7-day and 30-day all-cause mortality in a cohort of pelvic fracture patients and controls; Supplementary Figure S1: The Log (−Log) plots for assessment of proportional hazards in the age category of 50–64 years old; Supplementary Figure S2: The Log (−Log) plots for assessment of proportional hazards in the age category of ≥65 years old.
