# Supplementary material for: Pelvic Fractures—An Underestimated Problem? Incidence and Mortality Risk after Pelvic Fracture in Austria, 2010–2018
Source: J Clin Med. 2022 May 17;11(10):2834. doi: 10.3390/jcm11102834 (PMC9146576; doi:10.3390/jcm11102834)

Supplemental Table S1. Elixhauser comorbidities in a cohort of patients with pelvic fractures and their age-sex-matched fracture-free controls

	Pelvic fracture patients N (%)	Fracture-free controls N (%)	p-values
Congestive heart failure	12,315 (22.4)	5940 (17.8)	<0.001
Cardiac arrhythmias	17,083 (31.1)	8658 (25.9)	<0.001
Valvular disease	7075 (12.9)	3350 (10.0)	<0.001
Pulmonary circulation disorders	3839 (7.0)	1761 (5.3)	<0.001
Peripheral vascular disorders	7877 (14.3)	3791 (11.3)	<0.001
Hypertension, uncomplicated	32,943 (60.0)	17,452 (52.2)	<0.001
Hypertension, complicated	2845 (5.2)	1309 (3.9)	<0.001
Paralysis	825 (1.5)	268 (0.8)	<0.001
Other neurological disorders	6320 (11.5)	2252 (6.7)	<0.001
Chronic pulmonary disease	8896 (16.2)	3984 (11.9)	<0.001
Diabetes, uncomplicated	9019 (16.4)	4898 (14.7)	<0.001
Diabetes, complicated	5022 (9.1)	2596 (7.8)	<0.001
Hypothyroidism	5450 (9.9)	2234 (6.7)	<0.001
Renal failure	11,317 (20.6)	5190 (15.5)	<0.001
Liver disease	5144 (9.4)	2161 (6.5)	<0.001
Peptic ulcer disease, excluding bleeding	983 (1.8)	412 (1.2)	<0.001
AIDS/HIV	36 (0.1)	10 (0.0)	0.024
Lymphoma	913 (1.7)	411 (1.2)	<0.001
Metastatic cancer	2032 (3.7)	1520 (4.5)	<0.001
Solid tumour, without metastasis	7012 (12.8)	4957 (14.8)	<0.001
Rheumatoid arthritis/collaged vascular disease	2243 (4.1)	829 (2.5)	<0.001
Coagulopathy	2289 (4.2)	730 (2.2)	<0.001
Obesity	3328 (6.1)	2077 (6.2)	0.348
Weight loss	1954 (3.6)	677 (2.0)	<0.001
Fluid and electrolyte disorders	10,029 (18.3)	3758 (11.2)	<0.001
Blood loss anaemia	973 (1.8)	391 (1.2)	<0.001
Deficiency anaemia	3743 (6.8)	1343 (4.0)	<0.001
Alcohol abuse	2537 (4.6)	541 (1.6)	<0.001
Drug abuse	958 (1.7)	248 (0.7)	<0.001
Psychoses	691 (1.3)	268 (0.8)	<0.001
Depression	9444 (17.2)	3132 (9.4)	<0.001

Supplemental Table S2. Age and sex specific crude (CIR) and standardised incidence rates (SIR) per 100 000 of pelvic fracture in Austria between 2010 and 2018

**2010**	**MEN**				**WOMEN**				**TOTAL**				**IRR***
Age (Years)	No.population 2010	Pelvic fracture	CIR per 100.000	SIR per 100.000	No.population 2010	Pelvic fracture	CIR per 100.000	SIR per 100.000	No.population 2010	Pelvic fracture	CIR per 100.000	SIR per 100.000	
50-54	299702	169	56.4	378.8	302205	140	46.3	311.2	601907	309	51.3	344.8	
55-59	242329	163	67.3	384.9	253156	154	60.8	348.1	495485	317	64.0	366.1	
60-64	222252	158	71.1	345.9	237862	203	85.3	415.3	460114	361	78.5	381.8	
65-69	207704	208	100.1	432.3	234758	360	153.3	662.0	442462	568	128.4	554.2	
70-74	165923	230	138.6	555.3	197635	458	231.7	928.4	363558	688	189.2	758.1	
75-79	112078	204	182.0	561.8	155165	598	385.4	1189.6	267243	802	300.1	926.4	
80-84	79845	211	264.3	568.1	137500	888	645.8	1388.4	217345	1099	505.6	1087.1	
85-89	37712	159	421.6	598.0	101172	950	939.0	1331.8	138884	1109	798.5	1132.5	
90-94	8575	37	431.5	242.8	28004	267	953.4	536.6	36579	304	831.1	467.7	
≥95	2221	12	540.3	63.4	9310	79	848.5	99.5	11531	91	789.2	92.6	
Overall (≥50)	1378341	1551	112.5	125.3	1656767	4097	247.3	218.7	3035108	5648	186.1	185.4	1.7
**2011**	**MEN**				**WOMEN**				**TOTAL**				**IRR***
Age (Years)	No.population 2011	Pelvic fracture	CIR per 100.000	SIR per 100.000	No.population 2010	Pelvic fracture	CIR per 100.000	SIR per 100.000	No.population 2010	Pelvic fracture	CIR per 100.000	SIR per 100.000	
50-54	309695	181	58.4	392.6	310608	143	46.0	309.2	620303	324	52.2	350.9	
55-59	249804	181	72.5	414.7	260977	162	62.1	355.2	510781	343	67.2	384.3	
60-64	231426	185	79.9	389.0	248244	231	93.1	452.8	479670	416	86.7	422.0	
65-69	192127	202	105.1	453.9	217144	305	140.5	606.4	409271	507	123.9	534.8	
70-74	182343	256	140.4	562.4	215946	466	215.8	864.5	398289	722	181.3	726.2	
75-79	111044	216	194.5	600.4	151841	650	428.1	1321.4	262885	866	329.4	1016.9	
80-84	82450	221	268.0	576.3	135620	826	609.1	1309.4	218070	1047	480.1	1032.2	
85-89	39326	159	404.3	573.4	101586	874	860.4	1220.3	140912	1033	733.1	1039.7	
90-94	10365	62	598.2	336.6	33374	290	868.9	489.0	43739	352	804.8	452.9	
≥95	2022	10	494.6	58.0	8707	87	999.2	117.2	10729	97	904.1	106.1	
Overall (≥50)	1410602	1673	118.6	132.2	1684047	4034	239.5	213.7	3094649	5707	184.4	184.0	1.60

**2012**	**MEN**				**WOMEN**				**TOTAL**				**IRR***
Age (Years)	No.population 2012	Pelvic fracture	CIR per 100.000	SIR per 100.000	No.population 2012	Pelvic fracture	CIR per 100.000	SIR per 100.000	No.population 2012	Pelvic fracture	CIR per 100.000	SIR per 100.000	
50-54	320858	173	53.9	362.2	319817	133	41.6	279.3	640675	306	47.8	320.8	
55-59	259444	182	70.2	401.5	270177	156	57.7	330.4	529621	338	63.8	365.2	
60-64	228939	186	81.2	395.4	246410	204	82.8	402.9	475349	390	82.0	399.3	
65-69	191806	167	87.1	375.9	215973	292	135.2	583.7	407779	459	112.6	485.9	
70-74	194202	251	129.2	517.8	229812	520	226.3	906.5	424014	771	181.8	728.4	
75-79	111043	213	191.8	592.1	149589	608	406.4	1254.6	260632	821	315.0	972.4	
80-84	84012	238	283.3	609.0	134477	900	669.3	1438.8	218489	1138	520.9	1119.8	
85-89	41200	191	463.6	657.5	99603	892	895.6	1270.2	140803	1083	769.2	1090.9	
90-94	11945	65	544.2	306.2	38779	416	1072.7	603.7	50724	481	948.3	533.7	
≥95	1832	10	545.9	64.0	7988	79	989.0	116.0	9820	89	906.3	106.3	
Overall (≥50)	1445281	1676	116.0	129.9	1712625	4200	245.2	218.0	3157906	5876	186.1	185.7	1.7
**2013**	**MEN**				**WOMEN**				**TOTAL**				**IRR***
Age (Years)	No.population 2013	Pelvic fracture	CIR per 100.000	SIR per 100.000	No.population 2013	Pelvic fracture	CIR per 100.000	SIR per 100.000	No.population 2013	Pelvic fracture	CIR per 100.000	SIR per 100.000	
50-54	332435	159	47.8	321.3	330426	124	37.5	252.1	662861	283	42.7	286.8	
55-59	269216	178	66.1	378.4	279104	163	58.4	334.2	548320	341	62.2	355.9	
60-64	227365	172	75.6	368.1	244311	193	79.0	384.4	471676	365	77.4	376.6	
65-69	196227	198	100.9	435.6	220682	293	132.8	573.2	416909	491	117.8	508.4	
70-74	200794	262	130.5	522.7	237732	533	224.2	898.2	438526	795	181.3	726.3	
75-79	112706	212	188.1	580.6	149675	546	364.8	1126.0	262381	758	288.9	891.8	
80-84	84642	252	297.7	640.1	133426	888	665.5	1430.8	218068	1140	522.8	1123.9	
85-89	43200	169	391.2	554.9	97085	850	875.5	1241.8	140285	1019	726.4	1030.2	
90-94	13292	78	586.8	330.2	43491	449	1032.4	581.0	56783	527	928.1	522.3	
≥95	1700	13	764.7	89.7	7397	72	973.4	114.2	9097	85	934.4	109.6	
Overall (≥50)	1481577	1693	114.3	128.1	1743329	4111	235.8	210.4	3224906	5804	180.0	179.9	1.6
**2014**	**MEN**				**WOMEN**				**TOTAL**				**IRR***

Age (Years)	No.popula-tion 2014	Pelvic fracture	CIR per 100.000	SIR per 100.000	No.popula-tion 2014	Pelvic fracture	CIR per 100.000	SIR per 100.000	No.popula-tion 2014	Pelvic fracture	CIR per 100.000	SIR per 100.000	
50-54	342721	190	55.4	372.4	340200	134	39.4	264.6	682921	324	47.4	318.7	
55-59	280448	187	66.7	381.6	289394	179	61.9	354.0	569842	366	64.2	367.6	
60-64	227242	190	83.6	406.9	243847	196	80.4	391.1	471089	386	81.9	398.7	
65-69	199333	176	88.3	381.2	223415	293	131.1	566.2	422748	469	110.9	478.9	
70-74	199834	292	146.1	585.4	237317	570	240.2	962.2	437151	862	197.2	789.9	
75-79	123310	228	184.9	570.7	160411	642	400.2	1235.4	283721	870	306.6	946.5	
80-84	84581	260	307.4	660.9	131703	908	689.4	1482.2	216284	1168	540.0	1161.0	
85-89	45605	193	423.2	600.2	95820	945	986.2	1398.8	141425	1138	804.7	1141.3	
90-94	14363	86	598.8	337.0	47022	491	1044.2	587.6	61385	577	940.0	529.0	
≥95	1766	10	566.3	66.4	7516	67	891.4	104.6	9282	77	829.6	97.3	
Over-all (≥50)	1519203	1812	119.3	132.3	1776645	4425	249.1	222.9	3295848	6237	189.2	189.0	1.7
**2015**	**MEN**				**WOMEN**				**TOTAL**				**IRR***
Age (Years)	No.popula-tion 2015	Pelvic fracture	CIR per 100.000	SIR per 100.000	No.popula-tion 2015	Pelvic fracture	CIR per 100.000	SIR per 100.000	No.popula-tion 2015	Pelvic fracture	CIR per 100.000	SIR per 100.000	
50-54	350932	152	43.3	290.9	347843	138	39.7	266.5	698775	290	41.5	278.8	
55-59	292719	181	61.8	353.9	300051	157	52.3	299.4	592770	338	57.0	326.3	
60-64	231007	155	67.1	326.5	247760	213	86.0	418.4	478767	368	76.9	374.0	
65-69	205468	216	105.1	453.8	229354	291	126.9	547.7	434822	507	116.6	503.4	
70-74	185921	228	122.6	491.3	221689	499	225.1	901.7	407610	727	178.4	714.5	
75-79	141519	278	196.4	606.4	180927	679	375.3	1158.4	322446	957	296.8	916.1	
80-84	83856	261	311.2	669.1	129338	899	695.1	1494.3	213194	1160	544.1	1169.8	
85-89	47602	192	403.3	572.1	94475	877	928.3	1316.6	142077	1069	752.4	1067.2	
90-94	15216	85	558.6	314.4	48034	526	1095.1	616.3	63250	611	966.0	543.6	
≥95	2136	20	936.3	109.8	8902	82	921.1	108.1	11038	102	924.1	108.4	
Over-all (≥50)	1556376	1768	113.6	127.1	1808373	4361	241.2	216.2	3364749	6129	182.2	182.1	1.7
**2016**	**MEN**				**WOMEN**				**TOTAL**				**IRR***

Age (Years)	No.population 2016	Pelvic fracture	CIR per 100.000	SIR per 100.000	No.population 2016	Pelvic fracture	CIR per 100.000	SIR per 100.000	No.population 2016	Pelvic fracture	CIR per 100.000	SIR per 100.000	
50-54	356657	205	57.5	386.1	352827	142	40.2	270.3	709484	347	48.9	328.5	
55-59	303684	165	54.3	310.9	309201	181	58.5	335.0	612885	346	56.5	323.1	
60-64	239082	176	73.6	358.2	255956	195	76.2	370.7	495038	371	74.9	364.7	
65-69	214380	184	85.8	370.5	239313	326	136.2	588.1	453693	510	112.4	485.3	
70-74	171846	220	128.0	512.9	204933	438	213.7	856.2	376779	658	174.6	699.6	
75-79	155917	319	204.6	631.5	197790	741	374.6	1156.4	353707	1060	299.7	925.1	
80-84	83624	266	318.1	683.9	127262	912	716.6	1540.7	210886	1178	558.6	1200.9	
85-89	49501	214	432.3	613.2	93989	905	962.9	1365.7	143490	1119	779.8	1106.1	
90-94	16094	112	695.9	391.6	48611	518	1065.6	599.7	64705	630	973.6	547.9	
≥95	2556	20	782.5	91.8	10615	123	1158.7	135.9	13171	143	1085.7	127.4	
Over-all (≥50)	1593341	1881	118.1	132.0	1840497	4481	243.5	219.0	3433838	6362	185.3	185.3	1.7
**2017**	**MEN**				**WOMEN**				**TOTAL**				**IRR***
Age (Years)	No.population 2017	Pelvic fracture	CIR per 100.000	SIR per 100.000	No.population 2017	Pelvic fracture	CIR per 100.000	SIR per 100.000	No.population 2017	Pelvic fracture	CIR per 100.000	SIR per 100.000	
50-54	358346	188	52.5	352.4	355071	152	42.8	287.5	713417	340	47.7	320.1	
55-59	315477	202	64.0	366.4	318701	195	61.2	350.2	634178	397	62.6	358.3	
60-64	249027	185	74.3	361.5	265484	213	80.2	390.4	514511	398	77.4	376.4	
65-69	212572	193	90.8	392.0	237670	308	129.6	559.5	450242	501	111.3	480.4	
70-74	171619	235	136.9	548.6	203838	445	218.3	874.6	375457	680	181.1	725.6	
75-79	166022	305	183.7	567.1	210431	787	374.0	1154.4	376453	1092	290.1	895.4	
80-84	84346	234	277.4	596.4	125677	852	677.9	1457.5	210023	1086	517.1	1111.7	
85-89	50589	234	462.6	656.0	93570	902	964.0	1367.2	144159	1136	788.0	1117.7	
90-94	16972	110	648.1	364.7	47719	567	1188.2	668.7	64691	677	1046.5	588.9	
≥95	2861	24	838.9	98.4	11948	135	1129.9	132.5	14809	159	1073.7	126.0	
Over-all (≥50)	1627831	1910	117.3	130.6	1870109	4556	243.6	219.7	3497940	6466	184.9	185.1	1.7
**2018**	**MEN**				**WOMEN**				**TOTAL**				**IRR***

Age (Years)	No.population 2018	Pelvic fracture	CIR per 100.000	SIR per 100.000	No.population 2018	Pelvic fracture	CIR per 100.000	SIR per 100.000	No.population 2018	Pelvic fracture	CIR per 100.000	SIR per 100.000	
50-54	359192	192	53.5	359.1	355835	142	39.9	268.1	715027	334	46.7	313.8	
55-59	327395	210	64.1	367.1	329338	223	67.7	387.5	656733	433	65.9	377.3	
60-64	258948	210	81.1	394.6	274533	217	79.0	384.7	533481	427	80.0	389.5	
65-69	211529	194	91.7	395.9	235911	303	128.4	554.5	447440	497	111.1	479.5	
70-74	175755	248	141.1	565.3	208327	458	219.8	880.7	384082	706	183.8	736.4	
75-79	171171	347	202.7	625.8	217475	867	398.7	1230.6	388646	1214	312.4	964.2	
80-84	86342	267	309.2	664.8	126184	840	665.7	1431.2	212526	1107	520.9	1119.8	
85-89	51309	260	506.7	718.7	93144	961	1031.7	1463.3	144453	1221	845.3	1198.8	
90-94	17893	110	614.8	346.0	46741	553	1183.1	665.8	64634	663	1025.8	577.3	
≥95	3155	28	887.5	104.1	12948	156	1204.8	141.3	16103	184	1142.6	134.0	
Over-all (≥50)	1662689	2066	124.3	137.8	1900436	4720	248.4	224.7	3563125	6786	190.5	190.8	1.6

CIR Crude incidence rate; SIR standardized incidence rate; *Incidence rate ratio = IR women/IR men

Supplemental Table S3. Absolute number of patients with a diagnose of pelvic fracture, per fracture location

**Location of the fracture (ICD-10 code)**[1]	**Men**		**Women**	
	Absolute number	Mean age (SD)	Absolute number	Mean age (SD)
Os sacrum (S32.1)	1450	71.2 (12.0)	5811	78.1 (10.7)
Os coccyges (S32.2.)	127	70.5 (12.8)	343	75.9 (12.1)
Os ilium (S32.3)	592	69.9 (12.4)	792	78.9 (11.2)
Os acetabulum (S32.4)	2416	72.1 (12.2)	2463	79.3 (10.6)
Os pubis (S32.5)	4294	75.2 (11.7)	18,559	80.9 (9.6)
Os ischium (S32.6)[2]	1180	70.1 (12.3)	2553	78.0 (10.7)
Multiple fractures	997	68.3 (11.9)	1878	77.6 (11.1)
Fracture of lumbar spine and pelvis, without specified subcode (S32)	8429	70.7 (11.6)	15,624	76.3 (10.6)

[1] Sums of codes do not add up to the total number of pelvic fractures (n=54,975) because some patients had fracture on more locations of pelvis.
[2]Classification code for Os ischium in Austrian ICD-10 guidelines *(ICD-10 BMASGK 2020 – Systematisches Verzeichnis)* is S32.8

Supplemental Table S4. 7-days and 30-days all-cause mortality in a cohort of pelvic fracture patients and controls

	**Pelvic fractures**	**Controls**
**7-days mortality**	0.5% (n=256)	0.5% (n=254)
**30-days mortality**	2.0% (n=1056)	1.6% (n=895)

Supplemental Figure S1. The log(-log) plots for assesment of proportional hazards in age category 50-64 years old.

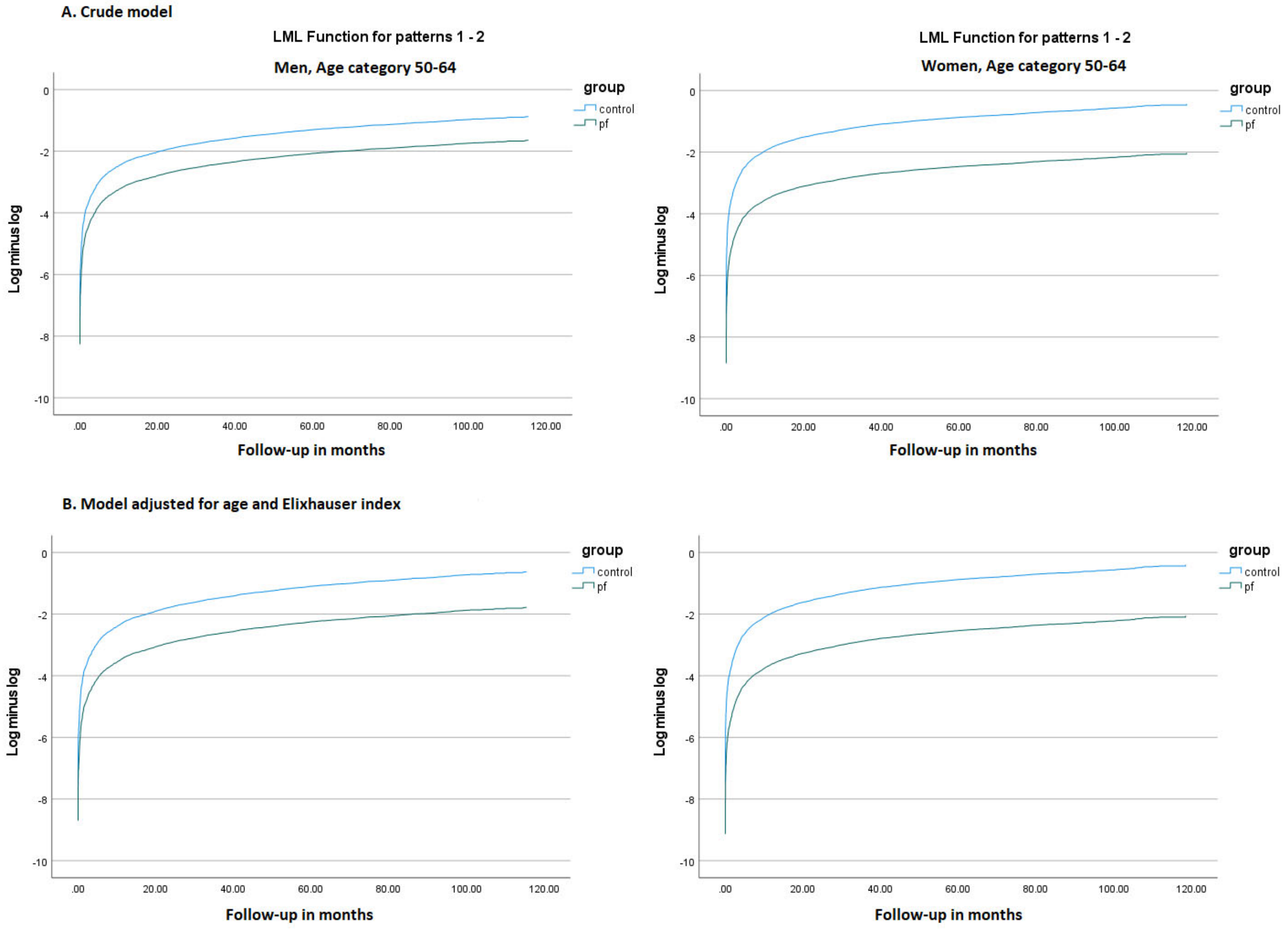

Supplemental Figure S2. The log(-log) plots for assesment of proportional hazards in age category ≥65 years old.

A. Crude model

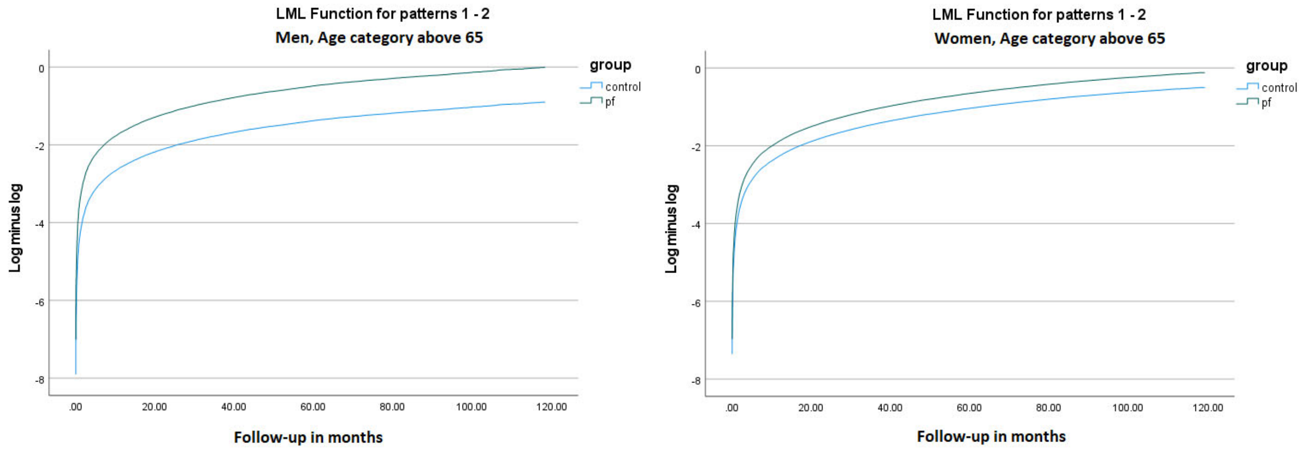

B. Model adjusted for age and Elixhauser index

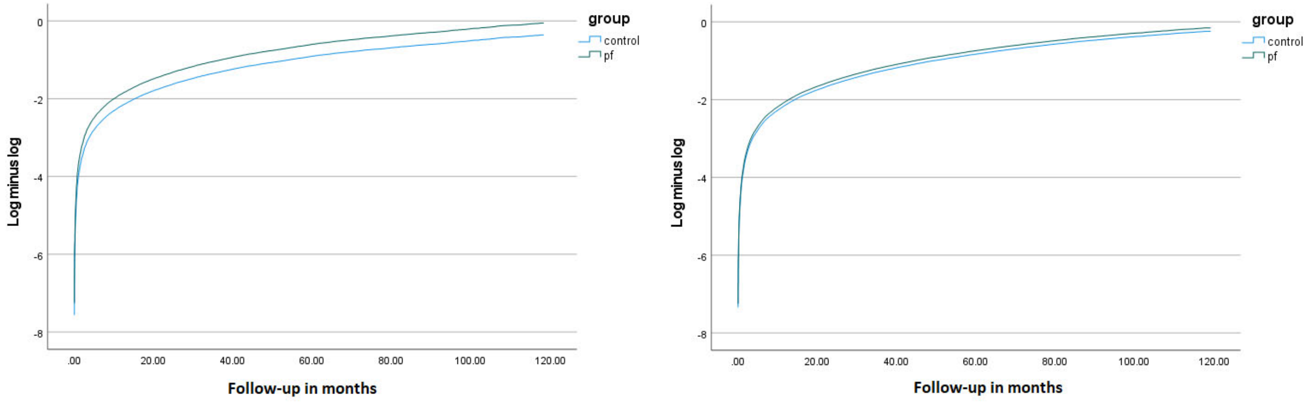

Supplement: Supplementary file 1 [file jcm-11-02834-s001.zip › jcm-1686808-supplementary.pdf]
